# Army Legislation

**Published:** 1893-11

**Authors:** 


*Write or see personally your Representative at Washington, and secure his support of this bill.*

---

Introduced in the Senate by Mr. Cameron.

Introduced in the House by Mr. Robinson.

Endorsed and recommended by Gen. Schofield and the War Department.

### AN ACT

TO FIX THE PAY, ALLOWANCES AND RANK OF THE VETERINARIANS OF THE UNITED STATES ARMY.

*BE IT ENACTED by the Senate and House of Representatives of the United States of America, in Congress assembled:*

SECTION I. That the pay of the United States Army Veterinarians shall be one hundred and twenty-five dollars ($125) per month, with the allowances, pensions, retirement, tenure of office and relative rank of a Second Lieutenant of Cavalry. *Provided*, however, that hereafter candidates for the United States Army Veterinary service shall comply with all the preliminaries now required from candidates for "The Army Medical Corps," and shall pass such examinations as the Secretary of War may direct.

SECTION II. This Act shall be in force and effect from and after its approval.

SECTION III. All Acts inconsistent with this Act are hereby repealed.

---

*Write or see personally your Representative at Washington, and secure his active support of this bill.*

